# Sustainability of a Non-pharmacological, Self-Managed Intervention for Chronic Musculoskeletal Pain: 3-group Randomized Controlled Pilot Trial

**DOI:** 10.21203/rs.3.rs-5314308/v1

**Published:** 2024-12-04

**Authors:** Yu-Min Cho, Chao Hsing Yeh, Huilin Wu, Xinran Huang, Wanqi Chen, Thomas J. Murphy, Jennifer Kawi

**Affiliations:** Cizik School of Nursing, University of Texas Health Science Center at Houston; Cizik School of Nursing, University of Texas Health Science Center at Houston; School of Public Health, University of Texas Health Science Center at Houston; School of Public Health, University of Texas Health Science Center at Houston; School of Public Health, University of Texas Health Science Center at Houston; Department of Family and Community Medicine, McGovern Medical School, University of Texas Health Science Center at Houston; Cizik School of Nursing, University of Texas Health Science Center at Houston

**Keywords:** Chronic Musculoskeletal Pain, Auricular Point Acupressure, Non-Pharmacological, Sustainability, Self-Management, Randomized Clinical Trial, Mobile Application

## Abstract

**Background:**

Chronic musculoskeletal pain (CMP) affects around 1.7 billion people globally, causing significant physical, psychological, and economic burdens. Current treatments often involve medications with risks, creating an urgent need for accessible alternatives. Interventionist-administered Auricular Point Acupressure (APA) has shown effectiveness in reducing pain. To explore whether this low-risk, non-invasive, non-pharmacologic, and non-opioid pain relief method can be expanded digitally, this study developed a self-managed APA program using mobile health technology and coaching videos, allowing individuals to self-administer APA and evaluate its impact and sustainability.

**Methods:**

A 3-group pilot randomized controlled trial was conducted among 37 participants with CMP. The participants were randomly assigned to in-person APA (n=14) with face-to-face APA instruction, self-guided APA (n=12) with remote APA coaching, or control (n=11). All received conventional healthcare, with the APA groups also receiving adjuvant APA intervention for a 4-weeks supplemented with mobile app coaching videos, followed by monthly monitoring for three months. Data on pain intensity, physical disability, physical function, anxiety, depression, fatigue, sleep disturbance, fear avoidance of work, and satisfaction were collected at baseline, immediately following the 4-week APA intervention, and during the 2-month and 3-month follow-ups post-intervention.

**Results:**

Both the in-person and self-guided APA groups experienced a 47% reduction in pain intensity. Over 50% of participants achieved at least a 30% reduction in pain, and more than 17% had a 30% reduction in physical disability. Improvements included an 18% boost in physical function, decreased fatigue, improved sleep disturbance, and less depression (with increases noted in the control group), a 50% reduction in fear-avoidance of work, and only 3.8% reported not being satisfied with the APA at the 3-month follow-up. No adverse effects related to APA were reported.

**Conclusion:**

APA improved physical and mental health in participants with CMP, enhanced readiness to return to work, and demonstrated sustainability for at least three months. Coaching videos delivered via a mobile app proved to be a feasible approach for teaching APA, increasing the accessibility of the intervention. This study highlights the impact of APA and recommends further research into its mechanisms and long-term benefits to support integration into standard practice.

## 1. Introduction

Chronic musculoskeletal pain (CMP) is a prevalent health issue and affects approximately 1.7 billion people globally [1]. It leads to physical, psychological, economic, and social implications, such as increased disability, insomnia, mental disorders, healthcareflexpenses, and public health concerns, as well as reduced quality of life and wellness [2]. The clinical treatment for CMP commonly includes prescription medications, physical therapy [3], exercise [4], and cognitive-behavioral therapy [5]. Medications for CMP such as nonsteroidal anti-inflammatory drugs (NSAIDs) [6], opioids [7], and adjuvants such as antidepressants [8], have resulted in physical and psychological improvements, but also involve potential risks or adverse effects, such as addiction, overdose, and effectiveness limitations [2]. Moreover, other modalities such as physical therapy and exercise are challenging for individuals with chronic pain [9], and cognitive-behavioral therapy may not be readily available or covered by insurance [10]. Therefore, identifying a pain relief method that is accessible, low-risk, cost-effective, non-invasive, non-pharmacologic, and free from the risks associated with opioids is crucial.

Auricular Therapy has shown effectiveness in delivering relief from pain for both acute and chronic conditions [11]. It provides relief for acute pain such as sore throats, low back pain, dysmenorrhea, and pain during infant blood draws. It also aids in perioperative pain control and alleviates chronic pain, including low back pain, musculoskeletal disorders, and headaches. Auricular Therapy, originating from Chinese medicine for over a millennium [12], gained modern scientific evidence in the 1950s through the studies of Paul Nogier [13, 14]. This therapeutic approach involves stimulating specific auricular acupoints, corresponding to various body parts, to restore energy balance, contribute to the healing process, and enhance overall well-being [14, 15]. Auricular therapy involves stimulating reflex points on the external ear using acupuncture, electroacupuncture, acupressure, lasering, cauterization, moxibustion, or bloodletting [12]. The most well-known auricular therapy, acupuncture, demonstrated pain reduction in cancer survivors with chronic musculoskeletal pain, however, participants ceased treatment due to ear pain, and there exists a potential risk for minor bleeding, bruising, infections, and other rare, serious complications [16–18]. Auricular Point Acupressure (APA), unlike acupuncture, provides a generally safe and needle-free alternative [17]. It has been shown to relieve pain by enhancing pain tolerance [19], demonstrating its high potential for managing pain.

Our earlier studies revealed that interventionist-administered APA yields improved outcomes in alleviating pain, including chronic low back pain [20, 21] and aromatase inhibitor-induced arthralgia [22]. The underlying mechanism of APA in mitigating chronic pain is through its anti-inflammatory pathway, inhibiting pro-inflammatory cytokines such as Tumor necrosis factor-alpha (TNF-α) and Interleukin 2 (IL-2) and augmenting the release of anti-inflammatory cytokines (e.g., IL-4) or β-endorphins [23]. Moreover, our functional magnetic resonance imaging (fMRI) results showed that APA stimulation may alter pain processing by changing the activity in the anterior cingulate cortex, dorsolateral prefrontal cortex, inferior parietal lobule, and insula for sensory, cognitive functions, and affective pain dimensions [24].

After demonstrating efficacy from our interventionist-administered APA producing positive outcomes in the alleviation of pain in our prior studies and amidst the Coronavirus Disease 2019 (COVID-19) pandemic, we developed a mobile Health (mHealth) approach using a mobile application for APA (mAPA) to facilitate a self-guided and self-managed implementation of APA intervention. In our longitudinal, one-group, open pilot study involving a 4-week APA intervention that utilized a mobile application for learning [25], we observed a significant 30% reduction in pain intensity and a noteworthy 47% improvement in physical function among patients with chronic musculoskeletal pain. Consequently, we customized the APA coaching videos and mobile application based on invaluable feedback from study participants, specifically tailoring them for individuals dealing with CMP to improve the implementation of the APA intervention. We subsequently conducted a randomized pilot trial to determine the impact and feasibility of mAPA for self-managing CMP.

Therefore, the aim of this study was to investigate the sustained impact of a needle-free, non-pharmacologic, and self-managed APA intervention for individuals with CMP. By developing a self-managed APA program using mobile health technology and coaching videos, we aimed to expand this low-risk, non-invasive, and non-opioid pain relief method digitally, evaluating its effectiveness and sustainability over a 3-month follow-up period, and extending our initial clinical observations.

## 2. Methods

### 2.1. Trial Design

This pilot, prospective, longitudinal, waitlist-controlled trial was carried out across multiple sites, encompassing two locations on the West and East Coasts of the United States. The research was approved by the Institutional Review Board (IRB) of Johns Hopkins University (IRB approval 00290512). The trial was preregistered on ClinicalTrials.gov (NCT03294148) and conducted from October 2021 to June 2022, with a 3-month follow-up. The protocol and detailed information on this randomized controlled trial has been published [26–28].

### 2.2. Participants

**Figure 1:**
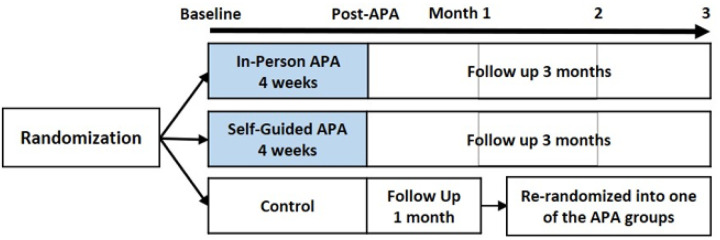


Eligibility criteria for participants included being 18 years or older, proficient in English reading and writing, experiencing CMP daily for at least 3 months or intermittently for most days of the week for at least 6 months with an average pain intensity of ≥ 4 on an 11-point scale in the preceding week, smartphone usage, and ability to effectively apply pressure to taped seeds on the ears. Exclusion criteria involved individuals with a latex allergy attributed to the tapes used for securing seeds on ear points. Participants were recruited, screened, and enrolled after providing informed consent, following an explanation of the study by trained research coordinators. Participants were randomly assigned to three groups using a computer-generated process based on simple randomization by the biostatistician. Only the project manager knew the group allocations, which were provided to a trained research coordinator at each site as participants enrolled in the study. However, the biostatistician and principal investigators for each site were blinded. Three groups were (1) in-person APA: received approximately 15-minute face-to-face APA instruction at the study site from the APA interventionist, supplemented with mAPA coaching videos, (2) Self-guided APA: received similar duration of APA coaching remotely with mAPA videos, and (3) Control: informed of their waitlisted status and after a 1 month waitlist period, were rerandomized into one of the APA groups to receive the APA intervention, giving them the chance to undergo the intervention post-waitlist. All participants received conventional healthcare and the two groups receiving adjuvant APA intervention for at least 4 weeks were then followed up monthly for three months (Figure 1).

### 2.3. APA Intervention

**Figure 2:**
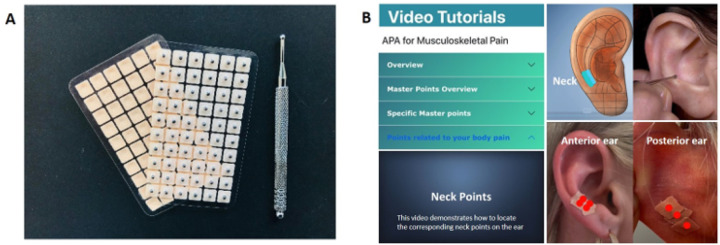
(A) The Vaccaria seeds with tape and the probe were used in the APA intervention. (B) The APA mobile application includes APA coaching videos, diagrams showing auricular acupoints and their corresponding painful body parts, question-and-answer sessions with APA instructions, and personalized visualization dashboards for self-monitoring and collecting participants’ self-reported outcomes.

The APA intervention involves preparing the skin with alcohol and locating tender acupoints using a probe. Vaccaria seeds, which are natural, non-toxic botanical seeds with no medicinal effects with external application [29] were then taped onto the desired acupoints, followed by manually applying pressure with the fingers to stimulate specific acupoints on the external ear (Figure 2A). The sensation of “da qi,” including soreness, tingling, numbness, energy awareness, mild electrical feeling, warmth, and mild throbbing often indicates correct seed placement and effective pressure [30, 31]. The APA groups were shown how to self-administer APA for 4 weeks (in-person or self-guided remotely with the mAPA) and self-managing this at home. The APA treatment lasted four weeks, with participants wearing the seeds for five days and removing them for two days before reapplying. The APA mobile application (Figure 2B) includes mAPA coaching videos, diagrams depicting auricular acupoints and their corresponding painful body parts, question and answer sessions with APA intervention instructions, and personalized visualization dashboards for self-monitoring of pain outcomes. Comprehensive details regarding the APA intervention can be found in our prior publications [25–27, 29].

### 2.4. Clinical Outcomes

Data were gathered at baseline, post-intervention (immediately after a 4-weeks of APA intervention), 2-month and 3-month follow-ups after the intervention. The primary outcome is pain intensity, while the secondary outcomes include physical and mental health, fear avoidance of work, and satisfaction with APA intervention. Data collection was conducted using Research Electronic Data Capture (REDCap), a secure, IRB-approved, and HIPAA-compliant data management system.

#### 2.4.1. The effects of APA on pain intensity

For the purpose of this study, we define CMP as pain that originates from the bones, muscles, or joints, such as pain in the neck, back, and joints (shoulder, hands, hip, knees, feet). Pain intensity was assessed through the self-reported Numeric Pain-Rating Scale (NRS) ranging from 0 to 10 with higher scores indicating greater pain. A decrease of 30% in pain intensity constitutes pain improvement, meeting the criteria for a “moderate clinically important difference” according to the Initiative on Methods, Measurement, and Pain Assessment in Clinical Trials (IMMPACT), and signifies a significant intervention effect [26, 32].

#### 2.4.2. The effects of APA on physical and mental health

Roland–Morris Disability Questionnaire (RMDQ) and Patient-Reported Outcomes Measurement Information System (PROMIS) 29 V2.0 were used for evaluating physical and mental function [33, 34] with validity, reliability, sensitivity, and substantial construct validity. A 30% improvement (reduction) from baseline in physical disability is considered a clinically meaningful change on the RMDQ [35]. PROMIS 29 V2.0, consisting of subscales for “physical function”, “anxiety”, “depression”, “fatigue”, and sleep disturbance, utilizes scores ranging from 4 to 20, with higher scores indicating more symptoms. Physical health was represented by the physical function in PROMIS 29 V2.0 and physical disability in RMDQ. Mental health was measured primarily by depressive symptoms and anxiety. Sleep disturbance and fatigue were also measured using the PROMIS 29 V2.0.

#### 2.4.3. The effects of APA on Fear Avoidance of Work

The “Fear Avoidance of Work” subscale of the Fear Avoidance Beliefs Questionnaire (FABQ) assesses an individual’s beliefs regarding work and its impact on their pain [36]. This 7-item screening tool employs a 0–6 Likert Scale to identify patients with high fear-avoidance beliefs, indicating a risk of prolonged disability. A lower score on this subscale suggests a higher potential for the individual to return to work.

#### 2.4.4. Satisfaction

Participants rated their satisfaction with the APA intervention using a self-report item, choosing from “not satisfied,” “somewhat satisfied,” or “completely satisfied [25, 27].

### 2.5. Data Analysis

Data analysis employed an intent-to-treat approach, including all enrolled participants regardless of treatment received, adherence, or withdrawal. Outcomes data for the control group after they were rerandomized to either treatment group were not included in the analyses to prevent any bias. Missing values for outcome variables were imputed using the “last value carried forward” method. Descriptive statistics were used to present demographic characteristics and study measures, with parametric analyses (means and standard deviations) applied to examine the outcomes. The analysis sample served as the denominator for all percentages, unless specified otherwise. Formal hypothesis testing was not performed due to the small sample size. Cohen classified effect sizes were used such as small (d = 0.2), medium (d = 0.5), and large (d ≥ 0.8) [37], where a positive value of Cohen’s d indicates the treatment group has a greater measured value than the control group, and a negative value indicates the treatment group has a lower mean than the control group [38]. Data analyses were conducted using SASand R 4.2.0.

## 3. Results

### 3.1. Participant Demographics

**Figure 3:**
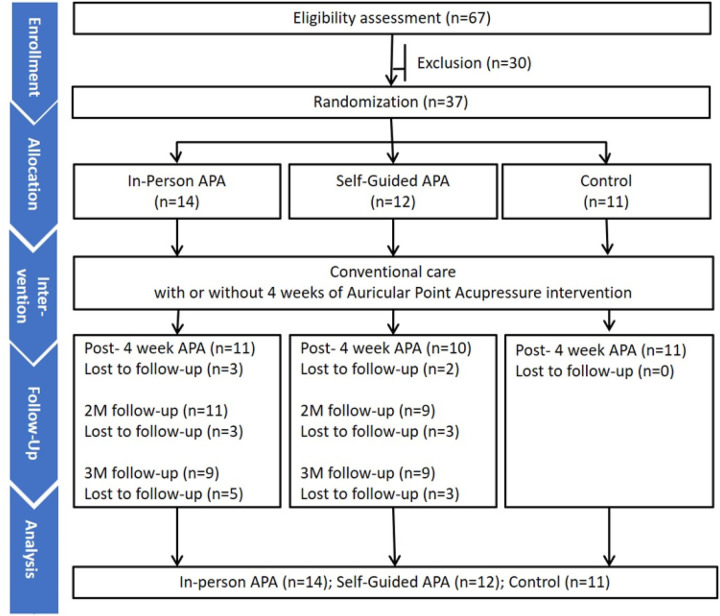
Consort diagram. APA: auricular point acupressure; 2M: 2-month follow-up after 4-week APA intervention; 3M: 3-month follow-up after 4-week APA intervention.

Thirty-seven participants met the study criteria and were enrolled in the study. They were randomized into three groups: in-person APA (n=14), self-guided APA (n=12), and waitlist control (n=11) (Figure 3). The average age of the participants was 50.5 years. Of these, 73% were female, 57% were White, and majority (84%) were not Hispanic. Many were college graduates (76%). The duration of CMP varied, with 43% experiencing pain for 1–5 years and 52% for more than 5 years. Most participants (79%) reported having musculoskeletal pain in three or more areas. The mean pain intensity at the primary pain location was 6.2 out of 10, with the back being the most common primary pain location (49%). At the 3-month follow-up, compared to the study’s start, more women (74%) than men (57%) completed the follow-up. Asian participants (75%) were more prevalent, while Black or African-American participants (50%) were the least to complete the 3-month follow-up. In terms of education, 67% of those with some college, 70% of college graduates, and 70% with post-graduate degrees completed the follow-up. The baseline demographic and clinical characteristics for each group are outlined in detail in our previous publication [26].

### 3.2. APA Decreased Pain Intensity

**Figure 4:**
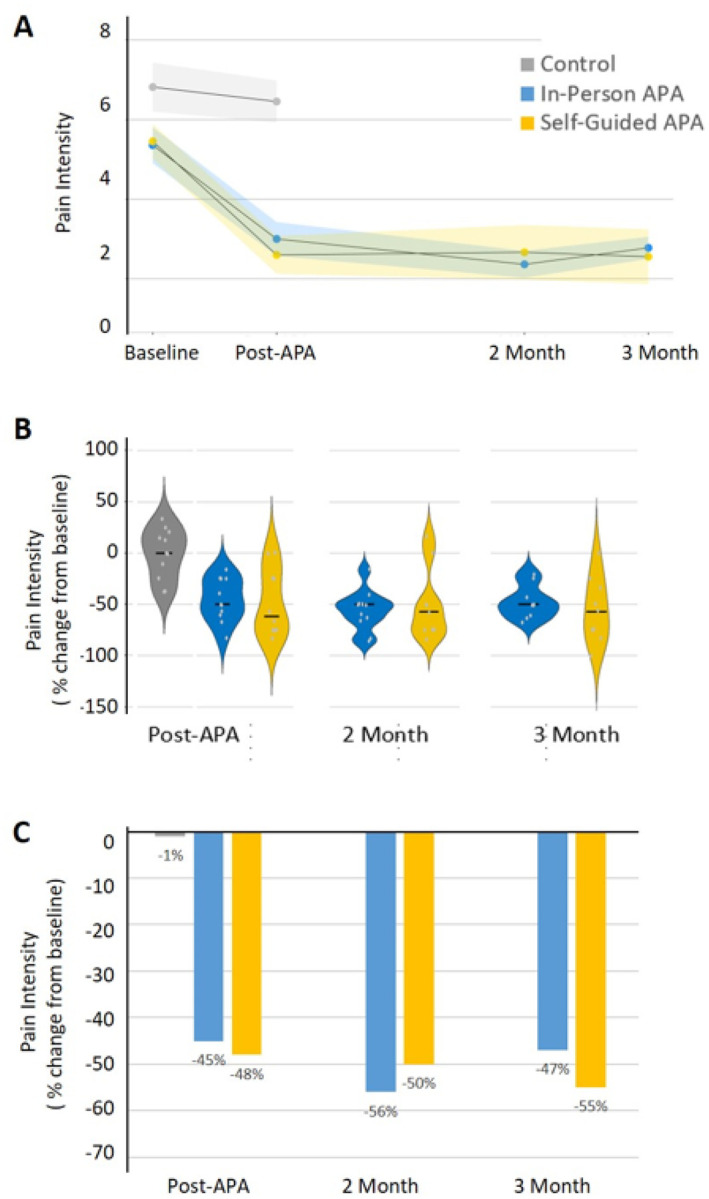
(A) Pain intensity was evaluated by NRS. Shading indicates standard error. (B) Percentage change in pain intensity from baseline to post-intervention, 2-month and 3-month follow-up. Dots represent individual participants; thick lines represent the group median. (C) The average percentage changes in pain intensity at post-intervention, 2-month, and 3-month follow-up. NRS: Numeric Pain-Rating Scale.

Figure 4 indicates that after 4 weeks of APA intervention, the percentage change in pain intensity for individuals showed a median reduction of 51% and 62% at immediate post-intervention, 50% and 57% at the 2-month follow-up, and 50% and 57% at 3-month follow-up in the in-person APA and self-guided APA groups, respectively (Figure 4B). The average change from baseline of pain intensity revealed a decrease of 45% and 48% at immediate post-intervention, 56% and 50% at the 2-month follow-up, and 47% and 55% at the 3-month follow-up in the in-person APA and self-guided APA groups, respectively, (Figure 4C). Significant differences in mean pain intensity were observed between the control group and the in-person APA group immediately post-intervention (Cohen’s d = −1.45), at the 2-month follow-up (Cohen’s d = −1.74), and at the 3-month follow-up (Cohen’s d = −1.53). Similarly, large differences in mean pain scores were noted between the control group and the self-guided APA group immediately post-intervention (Cohen’s d = −1.25), at the 2-month follow-up (Cohen’s d = −1.20), and at the 3-month follow-up (Cohen’s d = −1.54) (Table 1). Over 50% of the participants in both APA groups reached at least 30% reduced pain intensity after 4 weeks of APA intervention (Table 1), with the effects persisting at the 2-month and 3-month follow-ups. There was no significant difference in pain intensity observed between the in-person APA and self-guided APA groups after the same period. The control group had minimal changes in outcomes. Thus, APA intervention significantly decreased chronic musculoskeletal pain intensity, sustainable up to 3 months.

### 3.1. APA Improved Physical Health

**Figure 5:**
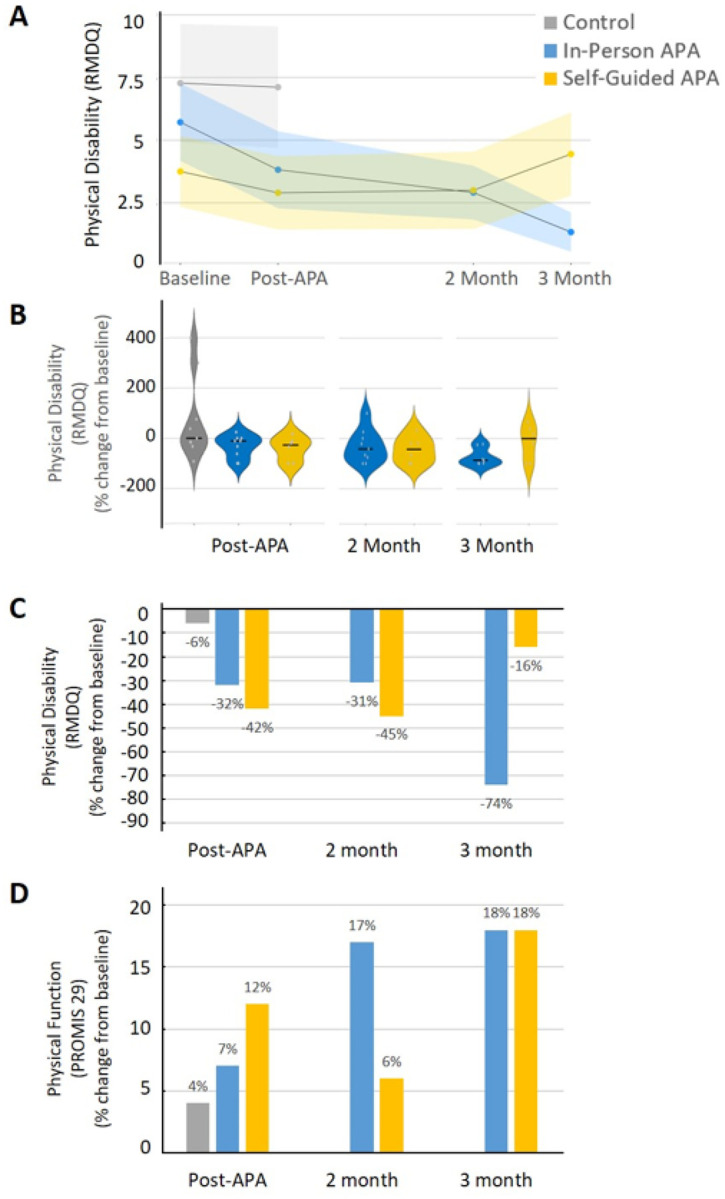
(A) Physical disability was evaluated by RMDQ. Shading indicates standard error. (B) Percentage change in physical disability from baseline to post-intervention, 2-month and 3-month follow-up. Dots represent individual participants; thick lines represent the group median. (C) The average percentage changes in physical disability at post-4 week of APA intervention, 2-month and 3-month follow-up. (D) The average percentage changes in physical function of PROMIS 29 V2.0 at post-4 week of APA intervention, 2-month and 3-month follow-up. RMDQ: Roland–Morris Disability Questionnaire; PROMIS: Patient-Reported Outcomes Measurement Information System.

Figure 5 indicates that after 4 weeks of APA intervention, the percentage change in physical disability showed a median reduction of 11% and 27% at immediate post-intervention, 42% and 43% at the 2-month follow-up, and 88% and 17% at the 3-month follow-up in the in-person APA and self-guided APA groups, respectively (Figure 5B). The average change from baseline of physical disability revealed a decrease of 32% and 42% at immediate post-intervention, 31% and 45% at the 2-month follow-up, and 74% and 16% at the 3-month follow-up in the in-person APA and self-guided APA groups, respectively (Figure 5C). Over 25% of the participants in both APA groups achieved at least a 30% improvement in physical disability after 4 weeks of APA intervention (Table 1). The control group showed minimal changes in outcomes. The average change from baseline physical function revealed an increase of 7% and 12% at immediate post-intervention, 17% and 6% at the 2-month follow-up, and 18% and 18% at the 3-month follow-up in in-person APA and self-guided APA groups, respectively (Figure 5D). Significant differences in mean physical function were observed between the control and in-person APA groups (Cohen’s d = 0.85), and moderate differences were observed between the control and self-guided APA groups at the 3-month follow-up (Cohen’s d = 0.65) (Table 1). Therefore, after the 4-week APA intervention, self-rated physical disability was reduced and physical function was enhanced in both the in-person and self-guided APA groups, with effects persisting at the 2-month and 3-month follow-ups.

### 3.4. APA showed promise in improving mental health

**Figure 6:**
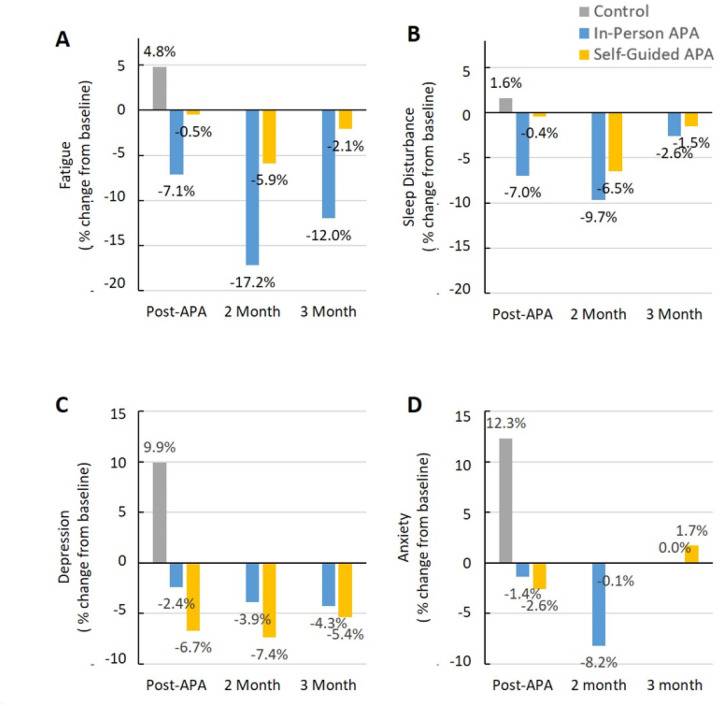
The average percentage changes from baseline in (A) Fatigue, (B) Sleep disturbance, (C) Depression, and (D) Anxiety based on PROMIS 29 V2.0 at post-4 week of APA intervention, 2-month and 3-month follow-up. PROMIS: Patient-Reported Outcomes Measurement Information System. Therefore, the APA demonstrated potential benefits in managing mental health by reducing fatigue, improving sleep quality, and alleviating depression symptoms, while the control group experienced increased depression and anxiety levels.

Figure 6 revealed the average percentage changes from baseline in mental health via PROMIS 29 V2.0 evaluation.

#### Fatigue:

The average change from baseline revealed a decrease of 7.1% and 0.5% at immediate post-intervention, 17.2% and 5.9% at the 2-month follow-up, and 12.0% and 2.1% at the 3-month follow-up in the in-person APA and self-guided APA groups, respectively. The control group showed an increase of 4.8% at immediate post-intervention (Figure 6A). Significant differences in fatigue were observed between the control group and the in-person APA group immediately post-intervention (Cohen’s d = −0.96), at the 2-month follow-up (Cohen’s d = −1.58), and at the 3-month follow-up (Cohen’s d = −1.08), while moderate differences in fatigue scores were noted between the control group and the self-guided APA group immediately post-intervention (Cohen’s d = −0.53), at the 2-month follow-up (Cohen’s d = −0.66), and at the 3-month follow-up (Cohen’s d = −0.53) (Table 1). As a result, the APA intervention may lead to a reduction in fatigue.

#### Sleep Disturbance:

The average change from baseline revealed a decrease of 7.0% and 0.4% at immediate post-intervention, 9.7% and 6.5% at the 2-month follow-up, and 2.6% and 1.5% at the 3-month follow-up in the in-person APA and self-guided APA groups, respectively. The control group showed a minimal increase of 1.6% at immediate post-intervention (Figure 6B). Moderate differences in mean sleep disturbance were observed between the control and in-person APA groups (Cohen’s d = −0.74) and between the control and self-guided APA groups at the 2-month follow-up (Cohen’s d = −0.60). However, the reduction in APA decreased- sleep disturbance was observed in both the in-person APA (Cohen’s d = −0.38) and self-guided APA groups at the 3-month follow-up (Cohen’s d = −0.20) (Table 1). As a result, the APA intervention may reduce sleep disturbances, leading to better sleep quality.

#### Depression:

The average change from baseline revealed a decrease of 2.4% and 6.7% at immediate post-intervention, 3.9% and 7.4% at the 2-month follow-up, and 4.3% and 5.4% at the 3-month follow-up in the in-person APA and self-guided APA groups, respectively. The control group showed an increase of 9.9% at immediate post-intervention (Figure 6C). Significant differences in mean depression were observed between the control group and the in-person APA group immediately post-intervention (Cohen’s d = −0.95), at the 2-month follow-up (Cohen’s d = −0.99), and at the 3-month follow-up (Cohen’s d = −1.09); it was also noted between the control group and the self-guided APA group immediately post-intervention (Cohen’s d = −1.27), at the 2-month follow-up (Cohen’s d = −1.29), and at the 3-month follow-up (Cohen’s d = −1.09) (Table 1). Thus, participants who received the APA intervention reported a reduction in depression symptoms, while the control group, which did not receive the intervention, showed an increase in depression levels.

#### Anxiety:

The average change from baseline revealed a decrease of 1.4% and 2.6% at immediate post-intervention, 8.2% and 0.1% at the 2-month follow-up, and 0% and an increase of 1.7% at the 3-month follow-up in the in-person APA and self-guided APA groups, respectively. The control group showed an increase of 12.3% at immediate post-intervention (Figure 6D). Significant differences in mean anxiety were observed between the control group and the in-person APA group immediately post-intervention (Cohen’s d = −1.07), at the 2-month follow-up (Cohen’s d = −1.23), and at the 3-month follow-up (Cohen’s d = −0.91). Similar differences were also noted between the control group and the self-guided APA group immediately post-intervention (Cohen’s d = −1.11), at the 2-month follow-up (Cohen’s d = −0.85), and at the 3-month follow-up (Cohen’s d = −0.76) (Table 1). Consequently, while the effect of the APA intervention on alleviating anxiety was inconsistent, the control group, which did not receive the intervention, reported an increase in self-reported anxiety.

### 3.5. APA Reduced Fear Avoidance of Work

**Figure 7:**
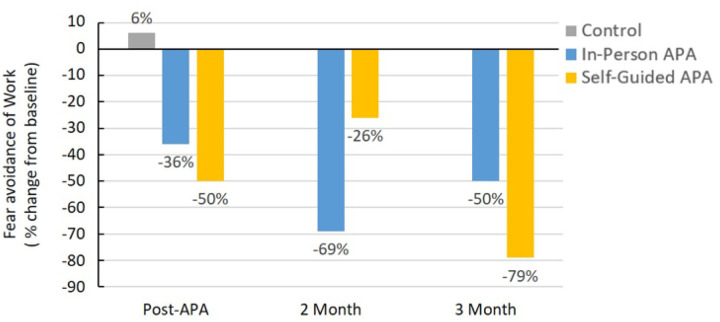
The average percentage changes from baseline in fear avoidance of work at post-4 week of APA intervention, 2-month and 3-month follow-up.

The in-person APA and self-guided APA groups showed average decreases from baseline in fear avoidance of work at 36% and 50% immediately post-intervention, 69% and 26% at the 2-month follow-up, and 50% and 79% at the 3-month follow-up, respectively. The control group exhibited an increase of 6% immediately post-intervention (Figure 7). Thus, APA can achieve a 50% reduction in fear-avoidance of work, suggesting that at least half of the participants who received the APA intervention felt ready to return to their jobs.

### 3.6. Satisfaction with APA intervention

**Figure 8:**
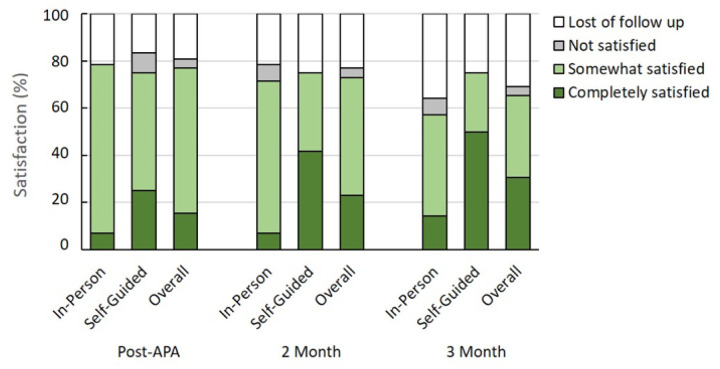
Self-reported satisfaction with the APA intervention

Figure 8 revealed participant satisfaction with APA intervention. In the in-person APA and self-guided APA groups, there were 79% and 76% at the post-intervention, 71% and 75% at the 2-month follow-up, and 67% and 75% at the 3-month follow-up who were “completely satisfied” or “somewhat satisfied”, respectively. The overall reported satisfaction with the APA intervention was approximately 65%. Lack of response was noted for 30.8% and only 3.8% indicated they were not satisfied.

## 4. Discussion

This prospective, longitudinal, randomized controlled trial demonstrated that adjuvant, self-managed APA can significantly reduce pain intensity in CMP and enhance both physical and mental health, with sustained effects lasting for at least three months after the 1-month intervention. The findings revealed that APA effectively lowered pain intensity and improved physical function. Furthermore, APA showed promising benefits for mental health by reducing fatigue, improving sleep quality, and alleviating depression symptoms, whereas the control group experienced worsening depression and anxiety. The APA intervention also led to a 50% reduction in fear-avoidance of work, indicating that half of the participants felt ready to return to their jobs, with 3.8% reported not being satisfied with the APA intervention. Additionally, no adverse effects related to APA were reported in this study cohort. Thus, these study findings are important due to the demand for sustainable and accessible, self-managed CMP interventions.

### 4.1. Participant Demographics

Results showed that females and college graduates were more likely to use adjunct interventions than males and high school graduates, aligning with previous studies [39–42]. Younger, female, Asian participants with some college education preferred to complete the adjunct APA interventions up to the 3-month follow-up. APA is a complementary and alternative medicine (CAM) treatment. Studies have reported inconsistent findings on the demographic characteristics of CAM users, with factors such as age, education, income, disease characteristics, urbanization, healthcare satisfaction, beliefs, support group attendance, and community networks influencing CAM use [39–44]. Although both genders use CAM to address ongoing health conditions and maintain wellness [45], young/middle-aged women focus more on improving their health and use CAM interventions more than men [39, 41, 46]. Studies indicate that Asian-Americans, influenced by a cultural emphasis on holistic Eastern medicine and exposure to both Eastern and Western health approaches, are more likely to use CAM and practice medical pluralism [47]. Individuals with higher education often seek CAM providers for additional healthcare options due to their higher income, ability to afford extra expenses, greater self-awareness, proactive health management, and enhanced knowledge and attitudes towards self-care, leading to better health and longer lifespans [48]. These are important variables to consider in future studies. Approaches and strategies to better target and retain male participants, individuals from diverse racial backgrounds, and those with lower educational backgrounds are essential for broader dissemination of APA in a diverse population. Emphasizing a culturally neutral approach and addressing educational disparities may enhance the intervention’s overall effectiveness and inclusivity.

### 4.2. APA Alleviated Pain Severity

APA can effectively alleviate pain. A systematic review and meta-analysis study indicated that auriculotherapy significantly reduces musculoskeletal pain in adults [49]. Additionally, performing APA twice a week for 10–15 minutes over four consecutive weeks significantly relieves chronic musculoskeletal pain in the spines of health workers [50]. This finding is consistent with our current and previous studies [26], which demonstrated that performing APA three times a day for three minutes per session, ve days a week for four consecutive weeks resulted in at least a 42% reduction in pain intensity; a 30% decrease in pain intensity signi es a signi cant improvement due to the APA intervention [26, 32]. Moreover, a self-guided APA intervention-induced pain relief similar to the in-person APA group for chronic musculoskeletal pain, with pain relief persisting for up to three months post-intervention.

### 4.3. APA Enhanced Physical Health

Self-managed APA can decrease CMP-induced physical disability and improve physical function. Participants who received a 4-week APA intervention experienced a 30% improvement in physical function, and over 25% of participants in the APA groups experienced more than a 30% reduction in physical disability; these improvements were observed even at the 1-month follow-up [26] and sustained up to 3 months follow-up. Similarly, overall disability level, pain intensity, and physical and functional abilities were observed to be significantly improved in elderly individuals with low back pain following auriculotherapy by using magnetic pellets [51]. Therefore, stimulating auricular acupoints might reduce pain, improve physical disability, and enhance physical and functional skills [51–53].

### 4.4. APA demonstrated potential in enhancing mental health

Self-managed APA demonstrated potential in improving mental health by reducing fatigue, enhancing sleep quality, and alleviating depression symptoms, while the control group, which did not receive the APA intervention, reported increased levels of depression and anxiety. Auricular therapy showed promise in alleviating chronic fatigue syndrome and cancer-related fatigue, with evidence supporting its efficacy and safety [54–56]. Auricular therapy can significantly enhance sleep quality. Auricular acupressure effectively reduces sleep disturbances and stress in middle-aged women, and similar benefits are observed in the elderly experiencing insomnia by using auricular acupuncture [53, 57–59], potentially reducing dependence on hypnotic medications [60]. Furthermore, Auricular therapy has shown benefits for depression, anxiety and stress [61, 62], including older adults in long-term care settings [63], in isolated COVID-19 patients [64], and health professionals [62]. Thus, auricular therapy provides a holistic approach to improving mental and physical well-being by addressing a range of symptoms through a valuable and non-pharmacological method across various populations.

### 4.5. Potential mechanisms of auricular therapy for alleviating pain and regulating mental health

The mechanisms for the use of Auricular Therapy for pain relief and mental health regulation are not fully understood. Based on the principles of Traditional Chinese Medicine, acupoints, where vital energy (qi) and blood flow from the internal organs and meridians reach the body surface, are thought to affect pain when obstructed [65]. This hypothesis was tested in mini pigs by injecting hydrogel, and auricular acupuncture near low hydraulic pressure points reduced nociceptive responses, supporting the meridianpain connection principle [66]. Stimulating auricular acupoints can regulate qi, restore the balance of yin and yang, and activate the body’s meridian systems, promoting natural healing and enhancing corresponding bodily functions [15, 67]. The mechanism behind this may be due to APA increasing anti-inflammatory cytokines (e.g., Interleukin-4, Interleukin-10) and endorphins while decreasing pro-inflammatory cytokines (e.g., Interleukin-2, TNF-α) and neuropeptides like Calcitonin Gene-Related Peptide (CGRP) [23, 68]. Stimuli at the ear’s nerve endings transmit signals to the central nervous system (CNS) via spinal and cranial nerves, where neurotransmitters modulate pain. This involves the descending neural pathway, which releases endorphins in the spinal cord to inhibit pain and balance sensory input. Stimulating auricular points can enhance mental health by activating the auricular branch of the vagus nerve, which boosts parasympathetic activity and reduces sympathetic nervous system activity, alleviating depression symptoms [69, 70]. This approach also stimulates the trigeminal nerve and transcutaneous vagus nerve, improving neuropsychiatric disorder treatment [71]. Additionally, auricular acupuncture may address serotonin depletion by inhibiting an overactive hypothalamic-pituitary-adrenal axis [72], further contributing to its mental health benefits [11]. Auricular therapy appears to support body homeostasis through mechanisms involving the autonomic nervous system, neuroendocrine system, neuro-immunological factors, neuroinflammation, and antioxidation [12], thereby promoting psychological and physiological regulation.

### 4.6. Mitigating Fear Avoidance of Work with APA

Chronic pain originates from a complex interplay of biological, psychological, and social factors, as viewed through the biopsychosocial perspective [73]. According to the fear-avoidance model, individuals who perceive pain as a threat often avoid activities they believe might exacerbate their condition. For instance, higher levels of pain-related fear have been linked to reduced physical activity among patients with low-back pain [74]. While such behavior can be adaptive in acute pain scenarios (e.g., allowing time for healing), prolonged avoidance of physical activities can impair daily functioning (e.g., reduced participation in work and leisure activities), increase negative emotions like depression, and contribute to greater disability due to physical deconditioning [75]. Longitudinal research has shown that individuals with low back pain who maintain a sedentary lifestyle experience higher levels of disability over time [76]. Our results indicated a decrease in fear avoidance of work among participants in both in-person and self-guided APA intervention groups, in stark contrast to an increase observed in the control group. These findings complement our other study results, indicating that APA effectively reduces pain, enhances impaired physical function, and alleviates symptoms of sleep disturbance, depression, and anxiety, with only 3.8% reported not being satisfied with the APA. Specifically, patients undergoing APA showed reduced fear avoidance of work, indicating a diminished perception of pain as a threat. This reduction in fear avoidance is crucial, suggesting that patients may be psychologically and physically prepared to return to work and resume productivity in daily activities. The ability to resume work not only improves their quality of life but also mitigates the socioeconomic impact of chronic pain. These outcomes highlight APA’s potential to facilitate the reintegration of individuals with chronic musculoskeletal pain into the workforce, addressing both personal and societal challenges.

### 4.7. Limitations

The nature of this pilot study precluded blinding, which may have influenced outcomes. The number of participants was limited; however, we were able to address multisite chronic musculoskeletal pain rather than focusing on a single pain location. Future studies with larger cohorts are needed to validate the effectiveness of APA in alleviating specific types of CMP.

To summarize, both in-person APA coaching and self-guided mobile APA improved pain relief, physical function, and mental health. With no significant differences between the two APA groups; mobile APA applications can effectively broaden the accessibility of APA interventions for chronic musculoskeletal pain management. This APA approach offers accessible, non-invasive, and non-pharmacologic pain relief, promoting self-management and empowering patients with an easily accessible, low-risk [16, 26], easy-to-implement, non-opioid alternative. Further studies should investigate APA’s mechanisms and their integration into multidisciplinary pain management programs, highlighting its potential as a cost-effective adjunctive therapy for improving quality of life and reducing medication dependency.

## 5. Conclusions

This non-invasive, self-managed, non-pharmacologic APA intervention improved both physical and mental health in participants with chronic musculoskeletal pain and may facilitate their return to work. Additionally, the APA intervention can be expanded digitally through the use of mobile applications and coaching videos, increasing its accessibility. Future research should explore APA’s mechanisms and long-term benefits for integration into sta ndard pain management practices.

## Figures and Tables

**Table 1:** Summary statistics for the study outcomes.

	In-Person APA(n = 14)	Self-Guided APA(n = 12)	Control (n = 11)	Cohen'sd		
				In-Person APA vs Control	Self-Guided APA vs Control	In-Person APA vsSelf-Guided APA
**Pain Intensity**						
Baseline	5.4 ± 1.7	5.5 ± 1.4	6.8 ± 2.0			
Post-APA	3.0 ± 1.4	2.6 ± 1.5	6.5 ± 1.8	−1.45	−1.25	0.09
≥30% reduction, n (%)	7 (50)	6 (50)	0 (0)			
2M follow-up	2.4 ± 1.1	2.7 ± 2.1	--	−1.74	−1.2	−0.27
≥30% reduction, n (%)	10 (71)	7 (58)	--			
3M follow-up	2.8 ± 0.8	2.6 ± 2.1	--	−1.53	−1.54	0.08
≥30% reduction, n (%)	7 (50)	7 (58)	--			
**Physical Disability**						
Baseline	5.7 ± 5.8	3.8 ± 4.9	7.3 ± 7.8			
Post-APA	3.8 ± 5.1	2.9 ± 4.6	6.0 ± 7.0	−0.03	−0.06	0.06
≥30% reduction, n (%)	4 (29)	3 (25)	0 (0)			
2M follow-up	2.9 ± 3.6	3.0 ± 4.6	--	−0.14	−0.07	−0.11
≥30% reduction, n (%)	5 (36)	3 (25)	--			
3M follow-up	1.3 ± 2.4	4.4 ± 5.0	--	−0.17	0.16	−0.63
≥30% reduction, n (%)	5 (36)	2 (17)	--			
**Physical Function**						
Baseline	39.0 ± 7.2	43.2 ± 5.8	38.3 ± 4.3			
Post-APA	41.7 ± 6.7	49.0 ± 6.0	39.9 ± 7.3	0.16	0.47	−0.32
2M follow-up	45.7 ± 7.2	46.0 ± 4.9	--	0.8	0.05	0.61
3M follow-up	46.3 ± 6.3	51.0 ± 7.2	--	0.85	0.65	−0.02
**Fatigue**						
Baseline	53.5 ± 10.1	54.7 ± 13.2	55.2 ± 11.2			
Post-APA	50.6 ± 8.2	53.5 ± 12.0	57.7 ± 13.3	−0.96	−0.53	−0.35
2M follow-up	45.0 ± 8.1	51.8 ± 14.6	--	−1.58	−0.66	−0.49
3M follow-up	46.8 ± 11.2	54.5 ± 13.6	--	−1.08	−0.53	−0.49
**Sleep Disturbance**						
Baseline	53.5 ± 6.8	50.3 ± 7.0	54.9 ± 8.0			
Post-APA	49.6 ± 4.5	51.8 ± 4.2	55.3 ± 7.4	−0.73	−0.14	−0.73
2M follow-up	48.1 ± 7.8	48.7 ± 5.4	--	−0.74	−0.6	−0.24
3M follow-up	51.8 ± 5.4	51.3 ± 6.9	--	−0.38	−0.2	−0.11
**Depression**						
Baseline	49.8 ± 7.5	49.6 ± 7.8	48.6 ± 7.8			
Post-APA	49.6 ± 8.6	46.3 ± 7.1	53.2 ± 10.7	−0.95	−1.27	0.49
2M follow-up	48.4 ± 6.5	45.2 ± 6.5	--	−0.99	−1.29	0.25
3M follow-up	48.1 ± 8.8	46.4 ± 8.1	--	−1.09	−1.09	0.14
**Anxiety**						
Baseline	51.0 ± 10.2	53.4 ± 12.0	51.6 ± 9.2			
Post-APA	51.1 ± 8.7	52.8 ± 11.9	57.5 ± 10.6	−1.07	−1.11	0.1
2M follow-up	47.2 ± 8.4	52.4 ± 10.3	--	−1.23	−0.86	−0.4
3M follow-up	50.3 ± 8.5	54.2 ± 12.8	--	−0.91	−0.76	−0.2
**Fear Avoidance of Work**						
Baseline	10.6 ± 16.4	14.7 ± 17.0	16.9 ± 21.1			
Post-APA	7.0 ± 10.6	12.1 ± 18.4	14.6 ± 11.5	0.18	−0.02	0.58
≥30% reduction, n (%)	3 (21)	4 (33)	0 (0)			
2M follow-up	5.1 ± 10.9	14.8 ± 19.4	--	0	0.17	−0.77
≥30% reduction, n (%)	6 (43)	1 (8)	--			
3M follow-up	2.9 ± 3.5	4.4 ± 10.1	--	0.18	−0.31	0.79
≥30% reduction, n (%)	3 (21)	4 (33)	--			

Data are presented as mean ± standard deviation. NRS: Numeric Pain-Rating Scale; APA: auricular point acupressure; RMDQ: Roland–Morris Disability Questionnaire; PROMIS: Patient-Reported Outcomes Measurement Information System; 2M: 2-month follow-up after APA the intervention; 3M: 3-month follow-up after the APA intervention; --: no value.

## Data Availability

Data from the current study are available from the corresponding author upon reasonable request.

## Abbreviations

APA: Auricular Point Acupressure
CGRP: Calcitonin Gene-Related Peptide
CMP: Chronic musculoskeletal pain
CNS: Central Nervous System
COVID-19: Coronavirus Disease 2019
FABQ: Fear Avoidance Beliefs Questionnaire
IL: Interleukin
IMMPACT: Initiative on Methods, Measurement, and Pain Assessment in Clinical Trials
IRB: Institutional Review Board
mHealth: mobile Health (mHealth)
NRS: Numeric Pain-Rating Scale
NSAIDs: Nonsteroidal anti-inflammatory drugs
PROMIS: Patient-Reported Outcomes Measurement Information System
REDCap: Research Electronic Data Capture
RMDQ: Roland–Morris Disability Questionnaire
TNF-α: Tumor Necrosis Factor-alpha
